# Feasibility and Acceptability of a Breastfeeding Support Intervention Among Mothers of Infants Under Six Months Old Discharged From Malnutrition Treatment in Kilifi County

**DOI:** 10.1111/mcn.70202

**Published:** 2026-05-14

**Authors:** Jackson Unda, Fridah Munene, Nancy Kagwanja, Caroline Jones, Martha Mwangome

**Affiliations:** ^1^ Kenya Medical Research Institute (KEMRI)‐Wellcome Trust Research Programme (KWTRP) Kilifi Kenya Kenya; ^2^ Oxford University – Nuffield Department of Medicine (NDM) Oxford UK; ^3^ Kenya Medical Research Institute (KEMRI)‐ Centre for Geographic Medicine Research (CGMRC) Kilifi Kenya

**Keywords:** breastfeeding peer supporters, Breastfeeding support, infant nutrition, infants under 6 months, malnutrition, wasting

## Abstract

Malnourished infants under 6 months experience poor growth and development. A pilot study applied the WHO guideline for treatment of acute malnutrition and found that it was possible to re‐establish exclusive breastfeeding during inpatient treatment. However, weight gain could not be sustained 6 weeks after discharge suggesting that follow‐up breastfeeding support may be required. Our study aimed to pilot the feasibility and acceptability of a follow‐up intervention among infants discharged from malnutrition treatment. A home‐based post‐discharge breastfeeding support intervention was developed to be delivered by breastfeeding peer supporters in a series of three home visits and three intervention phone calls over a period of 7 weeks after discharge from the hospital. The intervention was piloted among 20 mother‐infant pairs providing quantitative and qualitative data from a standardized questionnaire and in‐depth interviews. Data was analyzed descriptively and using thematic analysis. The median infant birth weight was 2.07 kg and > 50% of the mothers had primary education or less. The intervention was feasible with 100% reach of the target participants with a geographic spread of 25% urban and 75% rural areas. All intervention components were applied consistently, resulting in 100% adherence over a 6‐month period. The intervention was accepted by mothers, perceived to be beneficial, and reported to increase breastfeeding confidence. It was adaptable, adjusting to emerging challenges, and was successfully integrated into existing health services. The pilot demonstrated high feasibility and widespread acceptance of the intervention. Its effectiveness to improve weight gain among recovering infants is being determined in a trial.

## Summary

- Continued exclusive breastfeeding of infants under 6 months after discharge from malnutrition treatment will improve recovery and growth.
- The existing gap in guidelines and practice on follow‐up care of infants under 6 months discharged from malnutrition treatment indicates a need for a structured post‐discharge follow‐up support intervention to support recovery of these infants.
- A home‐based post‐discharge breastfeeding support intervention was developed to be delivered by breastfeeding peer supporters in a series of three home visits and three intervention phone calls over a period of 7 weeks after discharge from the hospital.
- The pilot demonstrated high feasibility and widespread acceptance of the intervention. Its effectiveness to improve weight gain among recovering infants is being determined in an on‐going clinical trial (PACTR202306789094184).

**Abbreviations**
BFPS: breastfeeding peer supporters
BFSI: breastfeeding support intervention
BKCall: booking call
CGMRC: Centre for Geographic Medicine Research Coast
CHP: Community Health Promoters
CRF: Clinical Report Form
EBF: exclusive breastfeeding
HV: home visit
IBAMI: improving breastfeeding among malnourished infants
INTVCall: intervention call
IQR: interquartile ranges
KEMRI: Kenya Medical Research Institute
LMIC: low‐ and middle‐income country
MUAC: Mid Upper Arm Circumference
NACOSTI: National Commission for Science, Technology and Innovation
SAM: severe acute malnutrition
SERU: Scientific and Ethics Review Unit
U6M: under 6 months
WAZ: weight for age Z score
WHO: World Health Organization
WLZ: weight for length Z score

## 1 Introduction

Severe acute malnutrition (SAM)/wasting, in infants under 6 months old (U6M), is a nutritional condition characterized by weight‐for‐length (WLZ) z‐scores <−2, and/or the presence of clinical signs of bilateral pitting edema (World Health Organization 2023). Globally, SAM affects millions of infants, with the highest number occurring in low‐ and middle‐income countries (LMICs). In 2021, the prevalence of SAM among u6m infants was estimated to be 24.5 million. This is a three times increase in prevalence when compared to 2011 when the estimate was at 8.5 million (Kerac et al. 2021). In Kenya, the demographic health survey of 2022 reports stunting and wasting prevalence of approximately 12% and 11.9% respectively among infants under 6 months (Government of Kenya GOK 2022).

Wasting in U6M infants leads to an elevated risk of childhood morbidity and mortality higher than that of wasted older children (6–59 months) (Kerac et al. 2011; Grijalva‐Eternod et al. 2017). The severity of malnutrition in this early infancy period influences the overall growth and development of these infants (Kerac et al. 2015) underscoring the need for timely and effective interventions for preventing, identification and management (World Health Organization 2023).

In 2013, the World Health Organization (WHO) released its first guidelines for the management and treatment of SAM in infants U6M. While these guidelines were updated in 2023 (World Health Organization 2023), the core recommendation for management of SAM in U6M infants remains the same: emphasizing the re‐establishment of exclusive breastfeeding (EBF).

Commonly, breastfeeding is known as a promotive intervention (Ochola et al. 2013; Horta et al. 2015; Kimani‐Murage et al. 2016; Kimani‐Murage et al. 2017; Kavle et al. 2019; Mituki‐Mungiria et al. 2020). However, for infants who are born nutritionally vulnerable, i.e. Preterm/Low Birth Weight (LBW) and or those who develop nutritional vulnerability after birth, EBF is a life‐saving nutritional intervention crucial for their treatment, recovery, growth, and survival (Ahmed and Sands 2010; Hamer et al. 2022; Khatib et al. 2023).

In Kenya, the Improving Breastfeeding among Malnourished Infants (IBAMI‐1) study piloted the implementation of the 2013 WHO guidelines using breastfeeding peer supporter (BFPS) and resulted in effective re‐establishment of EBF and infants attaining the recommended weight velocity among 65% of the enrolled infants after inpatient treatment (Mwangome et al. 2020) However, despite the intensive inpatient nutritional rehabilitation efforts, follow‐up data revealed a critical gap in sustaining adequate nutritional recovery and growth after transitioning to their homes without any form of structured follow‐up support offered post‐discharge (Van Ryneveld et al. 2020). The results highlighted an important existing gap in guideline and practice on follow‐up care of infants under 6 months discharged from malnutrition treatment indicating a need for a structured post‐discharge follow‐up support intervention for these infants.

To address this need, our research team used participatory methods involving interviews with stakeholders at national and county levels and a series of workshops with local practitioners to co‐design a home‐based post‐discharge breastfeeding support intervention (BFSI). The process of intervention development is described elsewhere (unpublished). It is the results from the piloting of the intervention evaluating its feasibility and acceptability that is the subject of this publication.

## 2 Materials and Methods

### 2.1 Study Design

The pilot study was conducted in Kilifi County Referral Hospital in Kenya and used a descriptive exploratory study design to assess the feasibility (applicability) and acceptability (administrable with minimum discomfort) of a home‐based post‐discharge BFSI.

### 2.2 Breastfeeding Support Intervention

The piloted intervention was a home‐based, individualized, face‐to‐face breastfeeding support provided by a peer supporter (BFPS) consisting of three home visits and three intervention calls over a period of 7 weeks after discharge from the hospital. Additional components included invitations of the area community health promoter (CHP), a breastfeeding partner (buddy) of the mother's choice to the home visit sessions with the aim of providing social support and linking the mother to other available community‐based psychosocial and health support systems.

#### 2.2.1 Intervention Follow‐Up: 3 Calls and 3 Visits

Within 48 h after discharge, BFPS made the first intervention call (IntvCall 1) which also served as booking call 1 (BkCall 1) for the first home visit. The call aimed to assess the mother/baby's progress after discharge and plan for the first home visit (HV1). The first home visit would happen within the first week after discharge and the BFPS would be accompanied by the CHP for this visit.

The second intervention call (IntvCall 2) and booking call (BkCall 2) were made in the second week after discharge and aimed to assess the mother/baby's progress since the last visit and plan for the second home visit 2 (HV2). HV2 happened in week four post‐discharge, and here the BFPS would plan to meet with the participant's breastfeeding buddy for information sharing and building breastfeeding support around the mother.

**Figure 1:**
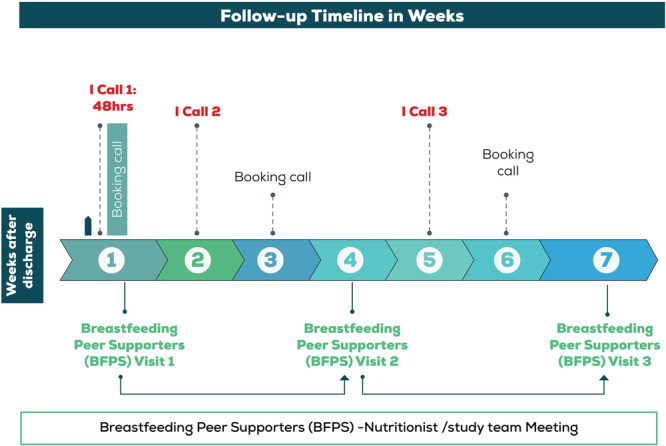
Components of the breastfeeding support intervention (BFSI).

The third intervention call (IntvCall 3) and booking call (BkCall 3) were done in the fifth‐week post‐discharge and aimed to assess the mother/baby's progress since the last visit and plan for the third home visit (HV3). HV3 was done in the seventh‐week post‐discharge and as this was the last home visit, the BFPS was accompanied by the CHP to support the transition (Figure 1).

During the home visits, the BFPS observe breastfeeding and correct positioning, attachment and suckling techniques of the infant to the breast. They also support mothers with hand expression where necessary and discuss practical strategies to continued EBF. They observe the infant and discuss danger signs while enquiring about infants' health. They also observe the mother for any signs of distress or overwhelm and their care practices such as hygiene and non‐verbal cues and may counsel the mothers where necessary.

### 2.3 Study Participants

#### 2.3.1 Inclusion Criteria

The primary study participants were infants aged between 4 and 12 weeks and their mothers admitted to Kilifi County te Referral Hospital. Infants were eligible if they were diagnosed with low anthropometry at admission, including a WLZ < −2 or a MUAC < 11.0 cm or a weight for age (WAZ) < −2, or the presence of bilateral pitting edema. Additionally, the study required that caregivers are willing and able to breastfeed the infants and consent to participate in the study.

Intervention implementers including breastfeeding peer supporters, field staff and managers were also included.

#### 2.3.2 Exclusion Criteria

Infants were excluded if they had congenital abnormalities that would obstruct breastfeeding, such as cleft palate, or had conditions that would invalidate the use of normal growth standards, such as hydrocephalus and other dysmorphic features. The pilot study opted to exclude pre‐term infants who had been hospitalized since birth and had never been discharged to go home as their mothers would not have had a home breastfeeding experience.

### 2.4 Sampling

A non‐probability sampling approach was used to purposively recruit the participants who met the inclusion criteria. A total of 20 dyads were enrolled in the pilot phase which was deemed sufficient, aligning with the recommendations for conducting a pilot study as a novice researcher, which suggests that 10–30 participants are adequate for gathering preliminary data (Doody and Doody 2015).

### 2.5 Data Collection

#### 2.5.1 Quantitative Data

Quantitative data was collected using a standardized questionnaire [case report form (CRFs)] administered at admission, discharge, and during the follow‐up period up to 6 months of age. The information captured in the CRFs mainly included mother and infant anthropometry and important demographic information such as age, level of education, marital status, and employment status and for infants age, gender, size at birth, place of birth and breastfeeding status. Data was sourced from both hospital child records and maternal recall. At admission, breastfeeding status was assessed by maternal recall however during admission and at discharge breastfeeding was assessed by observation and application of the WHO breastfeeding observation tool. Additional data was used to assess the feasibility aspect of the intervention for the key implementation indicators such as enrollment rates, adherence to the intervention, coverage/reach, fidelity and dropout rates.

#### 2.5.2 Qualitative Data

The researcher used semi‐structured interview guides tailored to conduct in‐depth interviews with the intervention recipients and implementers. The recipients' in‐depth interview content guide was focused on getting information on mothers' experiences, feelings and their views of the intervention to assess their acceptability of the intervention. While the implementers' in‐depth interview guide focused on their experiences, expectations, challenges, enablers and changes made during the implementation of the pilot intervention. A total of 12 in‐depth interviews were conducted with mothers in the national Swahili language: seven after the first home visit and five after the last. Each interview lasted between 20 and 25 min. Most mothers who participated in the first interview also took part in the second, except for two who had lost their babies.

Additionally, three interviews averaging 30 min were held with study implementers in Swahili language, including the BFPS (female), research officer(female), and field worker(male), after piloting the intervention.

### 2.6 Data Management

Quantitative data was initially entered into a REDCap ((Research Electronic Data Capture) database (Harris et al. 2009) and later transferred to Excel sheets, where it was cleaned to address any missing values and ensure accuracy. Data from all participants was included into the final analysis including data from infants who died before the end of the follow‐up period.

Interview recordings were transferred to a password‐protected computer for storage and then transcribed verbatim to ensure accuracy and preserve the richness of the participants' responses. Then, it was cleaned by listening to all the transcripts against the audio recordings to check on missing data and make corrections.

### 2.7 Data Analysis

#### 2.7.1 Quantitative Analysis

Descriptive analysis was done for the quantitative data focusing on the participants' demographic characteristics such as calculating the percentage, median, and interquartile range (IQR) using Microsoft excel. The feasibility constructs proposed by (Skivington et al. 2021), assessed metrics such as recruitment/enrollment, coverage/reach, adherence, fidelity and dropout rates (Table 1).

**Table 1:** Constructs of acceptability and feasibility.

Construct	Description
Feasibility	
Enrollment Rates	The number of participants enrolled in the intervention
Adherence	The degree to which participants follow the intervention protocol
Dropout Rates	The number of participants who discontinue the intervention before completion
Coverage/Reach	The proportion of the target population that the intervention reaches
Fidelity	The degree to which the intervention is delivered as intended
Adaptability	The ability of the intervention to be modified to fit the context of existing maternal/child health services
Integration	The extent to which the intervention is incorporated into existing maternal/child health services
Engagement Enablers	Factors that facilitate mothers' active participation and engagement with the intervention
Implementation Challenges	Challenges encountered during the delivery of the intervention
Implementers' Perceptions	The views and experiences of those who deliver the intervention, including their satisfaction and recommendations for improvement
Acceptability	
Perceived Effectiveness	The extent to which the intervention is perceived to achieve its purpose (perceived benefits)
Self‐Efficacy	The participants' confidence in their ability to perform behaviors required by the intervention
Affective Attitude	How the intervention makes participants feel.
Intervention Coherence	The participants' understanding of the intervention and its components.
Burden	The perceived effort required to participate in the intervention.
Ethicality	The fit of the intervention with participants' values and standards

#### 2.7.2 Qualitative Analysis

Thematic framework analysis was conducted following Braun and Clarke's six‐phase guide (Braun and Clarke 2023). This involved several steps: first, reading and re‐reading the transcripts to become thoroughly familiar with the data; next, generating initial codes to identify significant features within the data; then, searching for patterns among the codes to identify potential themes; reviewing the identified themes to ensure they accurately represented the data; defining and naming the themes, and finally interpretation and writing up the findings. Our interpretation of the themes identified were informed by the theoretical framework of acceptability (Sekhon et al. 2017). This framework consists of seven constructs: affective attitude, burden, ethicality, intervention coherence, opportunity costs, perceived effectiveness, and self‐efficacy(Sekhon et al. 2017) summarized in Table 1.

### 2.8 Ethical Considerations

The study was approved ‐ by the KEMRI Scientific and Ethics Research Unit (KEMRI/SERU/CGMR‐C/238/4326) and licenced by The National Commission for Science, Technology and Innovation (NACOSTI/P/23/24178). The participants were provided with detailed information about the study, and their consent was obtained before participation. The data collected was kept confidential and was used solely for this research.

## 3 Results

### 3.1 Participants Characteristics

The study involved 20 mother‐infant pairs.

The median age of the mothers at the birth of their first child was 22 years (IQR 19.75, 26 years), median weight, height and MUAC were 54.95 kg (IQR: 51.69, 67.70 kg), 152.1 cm (IQR: 149.6, 155.0 cm), and 280.0 mm (IQR: 240.5, 297.5 mm) respectively (Table 2).

**Table 2:** Maternal characteristics.

Characteristic (*N* = 20)	Median (IQR)
Mother's age at first child	22 (19.75, 26)
Anthropometric	
Weight	54.95 (51.69, 67.70)
Height	152.1 (149.6, 155.0)
MUAC	280.0 (240.5, 297.5)
Marital status	*N* = 20 (%)
Married	16 (80)
Single	3 (15)
Widowed	1 (5)
Education level	
Above secondary	1 (6.3)
Secondary	4 (25)
Primary	8 (50)
None	3 (19)
Unknown	4 (25)
Family setup	
Nuclear	9 (45)
Polygamous	1 (5)
Extended	8 (50)
Others	2 (10)
Employment status	
Casual/Irregular	5 (25)
Employed part‐time	1 (5)
No work income	5 (25)
Self employed	9 (45)

The median admission weight, length, MUAC and head circumference for infants were 2.295 kg (IQR: 1.87, 3.19 kg), 46.45 cm (IQR: 43.02, 51.42 cm), 86.0 mm (IQR: 74.0, 97.0 mm) and 34.25 cm (IQR: 32.55, 36.98 cm respectively (Table 3).

**Table 3:** Infant characteristics.

Characteristic	Median (IQR)
Anthropometric	
Admission Weight (Kg)	2.295 (1.87, 3.19)
Admission length (cm)	46.45 (43.02, 51.42)
Admission MUAC (mm)	86.0 (74.0, 97.0)
Admission head circumference (cm)	34.25 (32.55, 36.98)
Childbirth Weight (Kg)	2.065 (1.39)
Birth characteristics	*N* = 20 (%)
Infant twins (pairs)	3 (15)
Gender: Female	9 (45)
Birthplace	
Hospital	20 (100)
Delivery mode	
Cesarean	5 (25)
Vaginal	15 (75)
Gestation age	
Premature (< 37 weeks)	12 (60)
Term (> 37 weeks)	8 (40)

### 3.2 Feasibility Outcomes

As indicated earlier, feasibility was assessed using the framework approach proposed by (Husain et al. 2024) and (Skivington et al. 2021). Our results on feasibility are presented for each construct including reach, coverage, adherence, fidelity and intervention adaptability.

#### 3.2.1 Reach, Coverage and Adherence

The pilot study achieved 100% enrollment of its target participants within the stipulated time frame and accomplished a geographical spread of 25% and 75% in urban and rural areas respectively. All intervention components for the mothers in the pilot activities were completed on time, achieving a 100% adherence rate and a 0% dropout rate.

#### 3.2.2 Fidelity

The BFPS implemented the intervention components successfully, achieving 100% fidelity for Intervention Call 1, 95% for Call 2, and 80% for Call 3. In Home Visits, fidelity was 100% for Visit 1, 95% for Visit 2, and 85% for Visit 3. Strong family support engagement was noted, with 90% of mothers choosing a breastfeeding buddy, and 80% of those buddies being present during Home Visit 2. CHPs were linked to households at 100%, with 100% presence during Home Visit 1% and 80% during Visit 3.

#### 3.2.3 Intervention Adaptability

Continuous adjustments were made based on real‐time feedback and emerging challenges, during the intervention implementation.

##### 3.2.3.1 Infants' Recruitment

Initially, preterm infants who had never been discharged home since birth would be recruited into the study as soon as they became eligible, however during piloting an adjustment was made to only recruit infants presenting from home to maximize on the home experience.

It was seen that this mother had never gone home with the child since the child was born. So this child is not malnourished because of post‐discharge practice at home, but rather was just born malnourished because they were either preterm or they were very low birth weight or all these other things causing them to be born small… mostly born premature, and so what we did is we decided not to recruit these babies because the set of challenges that they have is quite different as opposed to those that, have already have been born either normal weight or had other challenges but have gone home and come back with malnutrition.

(IBM‐IDI‐ IMPL – 002)

##### 3.2.3.2 Booking Calls for Home Visits

The few mothers who were not reachable on the phone were contacted via their spouses' contacts which had been provided as secondary contacts.

##### 3.2.3.3 Transportation

Originally, the peer supporters were to use public transport to visit the households to maintain “peer‐ness” however, during the piloting this could not be arranged due to organization policies. Hence for the pilot phase, peer supporters used organization vehicles.

Yeah, the home visits were supposed to be done by breastfeeding peer supporters. They were supposed to use public means of transport, which means the same means of transport that the mother would choose to go to the homestead. We wanted to see how that would work in terms of how they're accessing the areas and using GPS to access those areas. Yeah, [but] according to the organisation's policy, an employee cannot use public transportation to the field, so now for the pilot we used the program vehicles. So that's one of the things that we changed for the intervention.

(IBM‐IDI—IMPL—002)

##### 3.2.3.4 Buddy Selection

Most mothers selected their husbands as breastfeeding buddies but many of them were unavailable during home visits. This was perceived to be a misunderstanding in the communication regarding the breastfeeding buddy component of the intervention. This finding led to a change in strategy on the timing and packaging of the breastfeeding buddy information. It was agreed to integrate the information of selection of buddies during hospitalization.

‘We were supposed to visit them at home, and the breastfeeding buddy was to be present if in case it was the husband or mother‐in‐law or something like that so we were preparing them early in advance that we needed the buddy present during the home visit and it should be someone who is readily available so in the pilot we were mainly dealing with post‐discharge but we decided that those who were coming to visit the mother while in the ward we would start talking to them that we would be visiting the mother at home in order for the mother to choose someone who would be really supportive and readily available’.

(IBM‐IDI‐ IMPL – 003)

##### 3.2.3.5 Mothers' Relocation

After the hospital discharge, some mothers opted to stay in relatives' homes instead of going back to their own homes. This was unexpected and it disrupted the home‐based follow‐up activities. An adjustment was made in the data capturing tools to allow for follow‐up of mothers as they relocated to new “homes.” For example, fresh mappings of homes were done for every new relocation.

##### 3.2.3.6 Infants Readmission

In cases where infants were readmitted back to the hospital, home intervention follow‐up would be temporarily frozen and later resumed upon discharge ensuring continuous home‐based support and follow up.

‘Because we had planned that there would be home visits and in between the home visits, the infant is readmitted, so an issue like this, we have to hold all the intervention activities because you cannot proceed with the intervention while the participant is readmitted in the facility so we had to hold all the activities and begin from where we left. We had not thought of that but because it happened we did not have any control, we just stopped the intervention and followed up later that was it’.

(IBM‐IDI‐ IMPL – 003)

#### 3.2.4 Intervention Integration to Maternal and Child Health Services

The involvement of community health extension workers (CHEWs^1^) helped to connect the CHPs with BFPS thus playing a significant role in integrating the intervention into the community health system.

‘Okay, I think that of CHPs because I think there was the issue of community engagement, so I think there was a very good community system because the CHP of that area we approached them by getting contacts from their chews and mostly XYZ got most of them and they accompanied her to the home visit and it was easier getting them through the contacts’.

(IBM‐IDI‐ IMPL – 003)

### 3.3 Acceptability Outcomes

Informed by constructs from the theory of acceptability framework (Table 1) (Sekhon et al. 2017) we identified six themes that indicated the overall acceptability of the intervention among the mothers. These themes are discussed in turn below.

#### 3.3.1 Mothers' Expectations of Intervention

Most mothers assumed the primary goal of the BFPS home visits was to discuss breastfeeding practices. However, a few expected additional support, such as financial aid or food donations, if they were deemed nutritionally vulnerable.

#### 3.3.2 BFPS Provided Useful Breastfeeding Information and Other Support

Interviews showed that peer supporters were a valuable resource for breastfeeding advice. Mothers successfully applied much of the guidance, such as proper breastfeeding positions and milk storage practices. However, some struggled with milk expression and felt overwhelmed by caregiving demands, making it challenging to follow feeding plans aimed at improving milk production.

‘Sometimes, here at home, there are many chores. First, when the baby cries, I have to sit and suckle it. I have to drink porridge according to my plan as I was given the frequency to drink porridge to stimulate milk production, but when I am about to drink the porridge, the baby cries, so I have to calm the baby, and when the baby is calm I realize even my schedule to drink porridge has already passed for I must ensure the baby is calm before I drink the porridge’

(IBM‐IDI‐HV1‐002)

#### 3.3.3 Predominant Positive Feelings About the BFPS

From the interviews, mothers felt comfortable communicating and interacting with the BFPS over the phone and in person. These interactions made it easier for them to discuss any challenges and get clarification as illustrated in the quote below:

‘Yes I got used to her [BFPS] I found out that she was a good mother and I could comfortably share any information with her and she could advise me accordingly and get more explanation.’

(IBM‐IDI‐HV2‐006)

### 3.4 Increased Confidence in Breastfeeding

Most mothers reported that they understood the advice from the BFPS and increased self‐confidence in breastfeeding their infants. Several mothers felt they were confident enough to educate other mothers who were unaware of the recommended breastfeeding practice. For example, one mother noted:

‘About how to position and hold the baby when breastfeeding, I was just positioning the baby anyhow provided she was breastfeeding and get satisfied, but I see now I have acquired a lot of knowledge, and I have become a teacher when I know another mother who is not positioning and holding the baby well I can comfortably tell her that is not how you hold the baby and I teach her how to hold it’.

(IBM‐IDI‐HV2‐007)

However, one mother in the home visit two interviews felt that it was difficult for her to grasp all the information because she was advised on multiple issues during the home visits.

#### 3.4.1 The Intervention Components Were Deemed Appropriate and Complementary to Each Another

From the interviews, the timing and duration of the calls and home visits were well‐suited to the mothers' routines. The home visits were reported to fit the mothers' schedules, except in one instance, where one mother found the timing of a home visit inconvenient. The BFPS had arrived very early in the morning before the mother had had breakfast. When the BFPS requested her to demonstrate how she was expressing milk, the mother felt uncomfortable and believed she couldn't produce any milk at that time.

‘She came very early in the morning, it was around 8. 00 am or 9.00 am, she told me to express milk but I told her I had not eaten anything but she insisted ‘just express and let me see the amount I could express. I did but I cannot remember the quantity of milk I expressed’.

(IBM‐IDI‐HV1‐007)

#### 3.4.2 Proposed Recommendations by Mothers

Mothers suggested several improvements to the intervention i.e. proposing the inclusion of questions about bed net use, family planning advice and provision of written materials for breastfeeding advice as illustrated below.

Aah, I can easily forget. It would be better if they could write information for me somewhere on paper that I can refer to later when she is not around or when I have forgotten.

(IBM‐IDI‐HV1‐006)

Mothers also suggested that the intervention be extended to other homes and that village‐wide meetings be organized to educate more mothers on breastfeeding practices. This suggestion was linked to the mothers' perspectives about how beneficial they found the intervention to be.

I don't know what you will say, but I thought all mothers should be informed about breastfeeding even if they are not breastfeeding because the information can help not only her but also maybe the grandchild.

(IBM‐IDI‐HV2‐002)

## 4 Discussion

The post‐discharge BFSI pilot study was found to be feasible by the implementers and acceptable by the recipient mothers of infants. The pilot study achieved successful recruitment and enrollment, thus being a good indicator of success of the laid‐out study procedures. Early success in recruitment, enrollment and retention of study participants reflects potential success in future recruitment when similar mechanisms are maintained (Walters et al. 2017). From our study, the successful recruitment observed indicates the success of the laid‐out procedures and boosts the confidence of similar replication in the main trial. The success in recruitment is not only a feasibility indicator but also an indicator of acceptability in the sense that it is associated with the perceived importance and benefit of the study by the participant (Bower et al. 2009).

Our findings agree with findings of a cluster randomized trial pilot study in rural India where complete participant inclusion through community efforts enhanced the intervention reach (Pérez et al. 2020). Our pilot study reached participants from both rural and urban areas which is a good measure of implementation coverage, supported by Clifford et al (Nkyekyer et al. 2021) and Goodrich et al (Ashcraft et al. 2024). If similar strategies are used there is promising replicability of the findings in the main trial. However, it is yet to be seen whether the plan for BFPS to use public transport to follow up at home will attain similar coverage.

The intervention demonstrated a high level of adherence to its components, reflecting high level of feasibility. Supported by Rempel, Rempel, and Moore (2017) and Rempel, Rempel et al. (2017), adherence to intervention components achieves better outcomes by maintaining a similar implementation plan. However, despite the observed level of adherence, infant death or mother's out‐migration to non‐study geographic zones may affect adherence levels in the main trial which intends to follow‐up infants up to 12 months of age.

The intervention can be practically and easily implemented as demonstrated by the fidelity findings. The success of replicating similar implementation fidelity in the main trial is supported by a conceptual framework (Carroll et al. 2007), that emphasizes the importance of maintaining high fidelity for the success of intervention implementation.

The pilot intervention encountered some challenges during its implementation phase. The pilot, however, consistently adapted to challenges adjusting throughout the process. For instance, a few mothers who were not reachable on phone, were later contacted via their spouses' contacts which had been provided as secondary contacts.

Our study findings were in line with the findings from other studies (Eldridge et al. 2017) which indicate that successful interventions are those that can adapt to local needs. Furthermore, flexibility and adaptability are essential in community‐based breastfeeding support programs to address unexpected challenges and improve outcomes.

The pilot study demonstrated significant potential for the intervention to be integrated into the existing maternal and child health services within the healthcare system. As revealed by the results, the CHPs played a crucial role in providing ongoing community support and ensuring the sustainability of breastfeeding even after the study was completed. Other studies have yielded similar results, indicating that involving CHPs in health interventions significantly enhances sustainability and ensures continuity over time (Perry et al. 2014; Kok et al. 2015; Vaughan et al. 2015). This is because CHPs are at the base of the health system and are best suited to bridge the gap between the health care system and the community.

On acceptability, all mothers expected the intervention to provide support on breastfeeding, showing their understanding of the intervention's purpose. According to (Sekhon et al. 2017), the mothers' expectations aligned well with the Intervention Coherence construct suggesting that the intervention was well‐understood. Acri et al (Acri et al. 2015) argue that well‐understood interventions are likely to be well‐received, an outcome supported by the findings from our study. Though some mothers anticipated other forms of support, this is a normal observation as it is expected that there are varied expectations for individuals participating in any intervention (Rollins et al. 2016).

Mothers found the intervention to be effective, reporting that they gained valuable knowledge on breastfeeding, which they successfully applied to improve their practices. However, some mothers also mentioned that household chores added to their demands, making it challenging to implement all the knowledge they had acquired. Despite these challenges, our findings indicate that the intervention was highly acceptable to the mothers, as evidenced by their positive reception of the advice and their commitment to follow it. Several studies (McFadden et al. 2017; Clarke et al. 2020), have demonstrated that positive responses of the participants towards the intervention are indicative of a high level of acceptability of an intervention. This alignment suggests that the designed intervention was highly valued and received widespread recognition based on the perceived and demonstrated benefits.

There were high levels of breastfeeding confidence observed among the mothers which indicated a high level of self‐efficacy obtained through their participation in the intervention. The knowledge received from the BFPS was reportedly eye‐opening to the mothers with several of them wishing they had the information before having their other children.

The significance of self‐efficacy in the effectiveness of breastfeeding interventions is supported by others who proposed that interventions focusing on enhancing mothers' confidence yield positive results and are well‐received. Similarly, (Otsuka et al. 2014), concluded that mothers who have faith in their ability to breastfeed and receive consistent support demonstrate high acceptance levels.

Most mothers widely praised their interactions with the BFPS, demonstrating a positive feeling about the BFPS support and the intervention. This indicates that the continuous follow‐up and support by the BFPS improved their interactions with the mothers, leading to increased positive feelings towards them and the intervention. This finding is consistent with (McFadden et al. 2017), who emphasized the importance of ongoing follow‐up support in improving the acceptability of interventions and fostering positive attitudes towards the intervention. Others have also confirmed that regular interaction increases confidence and builds trust, leading to higher satisfaction, and acceptance of an intervention.

BFPS tackled most tasks, leaving mothers with simple routines and techniques to follow, such as proper breastfeeding positioning. Most mothers reported to have received the intervention well, as it required minimal effort from them to follow the easy and straightforward routines. This finding is in line with other studies indicating that when mothers are provided with clear and easy‐to‐follow practices and instructions that demand minimal effort, they are more likely to comply with and accept the intervention.

### 4.1 Limitations

We acknowledge that the findings are from a pilot study with a small sample size and hence generalizability is limited. The pilot failed to utilize public transportation for the BFPS due to the organization policy that necessitated the use of organization vehicles even though the study team will use public vehicles in the main trial. The acceptability and feasibility of this approach to use public transport may need to be assessed. Use of public transport for the peer supporters helps to maintain their peer‐ness during follow‐up visits which is fundamental to gaining trust and openness from the mother.

### 4.2 Recommendations

For the main trial, it is essential to incorporate additional questions on intervention calls, including family planning topics during home visits and provide written information and communication (IEC) materials containing breastfeeding information.

## 5 Conclusion

From our findings, we can ascertain that the BFSI intervention is feasible and acceptable among mother‐infant pairs, allowing for the main trial to progress. Enhancing peer‐ness of the supporters, for example by using public transport during follow‐up visits will further improve acceptability of the intervention. Recommendations from the mothers for example on additional topics informed trial training curriculum for the BFPS. Findings from the main trial will provide evidence for effectiveness of a structured post discharge follow‐up strategy for infants recovering from malnutrition.

## Author Contributions

M.M. and C.J. conceptualized the study and applied for funding. M.M., C.J. and F.M. developed the intervention and implemented the pilot study. M.M., F.M. and N.K. supported J.U. in analysis and write up of the data. All authors read and approved the final manuscript.

## Conflicts of Interest

The authors declare no conflicts of interest.

## Data Availability

The data that support the findings of this study are available from the corresponding author upon reasonable request.

## Endnotes

1 A CHEW is a health provider based at a health facility who supervises CHPs providing health services in the community.
